# Classification of Variable Foundation Properties Based on Vehicle–Pavement–Foundation Interaction Dynamics

**DOI:** 10.3390/s20216263

**Published:** 2020-11-03

**Authors:** Robin Eunju Kim

**Affiliations:** Department of Civil and Environmental Engineering, Hanyang University, Seoul 04763, Korea; robinekim@hanyang.ac.kr; Tel.: +82-2220-0413

**Keywords:** machine learning-based classification, non-uniform foundation, stochastic analysis, vehicle–pavement–foundation interaction

## Abstract

The dynamic interaction between vehicle, roughness, and foundation is a fundamental problem in road management and also a complex problem, with their coupled and nonlinear behavior. Thus, in this study, the vehicle–pavement–foundation interaction model was formulated to incorporate the mass inertia of the vehicle, stochastic roughness, and non-uniform and deformable foundation. Herein, a quarter-car model was considered, a filtered white noise model was formulated to represent the road roughness, and a two-layered foundation was employed to simulate the road structure. To represent the non-uniform foundation, stiffness and damping coefficients were assumed to vary either in a linear or in a quadratic manner. Subsequently, an augmented state-space representation was formulated for the entire system. The time-varying equation governing the covariance of the response was solved to examine the vehicle response, subject to various foundation properties. Finally, a linear discriminant analysis method was employed for classifying the foundation types. The performance of the classifier was validated by test sets, which contained 100 cases for each foundation type. The results showed an accuracy of over 90%, indicating that the machine learning-based classification of the foundation had the potential of using vehicle responses in road managements.

## 1. Introduction

Road infrastructure forms a basic component in transportation, providing connectivity between local, regional and global value chains. Despite the impacts of road’s serviceability on the economy and public safety, maintenance is inadequate, due to its extensive nature. For example, the Federal Highway Administration reported that 26 percent of major urban roads in the U.S. are in a poor condition [1]. FHWA also reported that a capital of 182 billion dollars was spent in 2008 on improvements and maintenance of federal highway, while they are still in shortage [1]. Thus, research that develops tools and methods for assessing road conditions assume greater importance.

Typically, a pavement’s condition is assessed by measured information, such as ride comfort, surface defects, and structural adequacy [2]. For example, the Pavement Condition Index (PCI), developed by the U.S. Army Corps of Engineers rates the surface operational conditions including rutting, potholes, crackings, etc. [3]. Recent advances in image processing and deep learning technologies demonstrated that an automatic rating of PCI using a visual platform is available. For example, the automated pavement management system equipped with visual inspecting tools were developed to evaluate the pavement deteriorations and cracking [4,5]. Multimetric sensors including wireless sensing modules are utilized as well to examine the surface condition [6,7], fatigue [8], and certain anomalies on road structures [9]. However, unlike visible surface defects, structural adequacy is associated with the load transfer capability of the subgrade layers.

The falling weight deflectometer (FWD) is an essential non-destructive tool that is widely used for evaluating the structural adequacy of the pavement [10]. In FWD, as the weight falls on the pavement to be examined at some height, the response of the pavement, including deflection, is measured. Then, the responses are related to the strain and elasticity of the pavement, to examine the adequacy of the sublayer [11]. However, due to complexities in the testing method, which is usually performed during post-processing of the collected data, and due to required technical expertise, significant time, and costs, the network-level application is limited [12]. With the advancement of the wireless module’s sensing and calculating capability, the inspection and monitoring fields are in the transition from human-oriented inspection to machine-based inspection [13]. The following literature shows some successful examples of monitoring foundation noise excitation [14], decentralized road networks [15], which are known to be complicated, compared to other applications. Thus, so far, predicting the capability of road structure with rather portable and automated devices are of interest, but a challenging task, due to its complicated mechanisms.

Within a road structure, the main excitation source is a moving vehicle. To understand the responses of the moving loads, various foundation types were examined, based on analytical models. One of the simplest model was developed to examine vehicle response due to road roughness on a non-deformable foundation. Roughness was first modeled as Gaussian random signals [13]. Then, the models improved to contain a more realistic input, such as a stationary zero-mean process with a certain power spectral density (PSD) [16,17,18]. Among various research works, Wedig derived a closed-form expression of the covariance response of a vehicle model, by integrating the PSD of road roughness [19]. These models examined the impact of road roughness on vehicle responses, while the interactions due to pavement deflection were neglected.

To consider the deformable foundation, an Euler-Bernoulli beam resting on the viscoelastic foundation was investigated. Hardy and Cebon (1993) developed a quarter-car model on a smooth beam on a uniform Winkler foundation, to examine the vehicle–pavement–foundation interaction [20]. Similar approaches were adopted by other researchers and they used the models to understand the impact of vehicle parameters (including speed) on foundation responses [18,21]. Kelvin foundation under the Bernoulli beam was also adopted by authors in [22]. In their model, the interaction responses were examined by coupling the solutions of two systems—(1) vehicle on rough road and (2) elastic foundation subject to a single load. To eliminate the boundary condition effects, the frequency domain analysis of the interaction problem was performed on an infinite length beam [23]. Instead of handling infinite length, Kim et al. (2019) formulated the interaction system, based on a moving coordinate system, and examined the second-order stationary response of the interaction problem [24]. In the aforementioned studies, the foundation properties such as stiffness and density were assumed to be uniform, while in reality, those quantities might vary along the length of the road.

The non-uniform foundation on a beam was investigated by several groups of researchers. Early efforts focused on formulating a closed-form equation for varying foundation modulus, targeting statistical analyses. The linearly varying solutions were presented by Franklin and Scott (1979) [25] and higher-order variations were solved by the authors in [26,27]. The free vibration of the beams on the non-uniform foundation was studied by the following authors [28,29,30]. The authors in [30] compared the impact of nonlinear foundation on the deflection shapes and natural frequencies of the beam. Then, dynamic responses of a beam on the variable Winkler foundation, subject to a moving load, were studied by [31,32,33], and a moving mass was investigated by [34]. Although previous studies captured the effect of variable foundation on the pavement system, due to computational complexities, studies mostly neglected the inertial force effect from the moving vehicles.

In this study, the impact of the non-uniform foundation on vehicle responses was solved by developing the vehicle–pavement–(non-uniform)-foundation interaction model. In the model, the vehicle was represented with a moving-oscillator (a quarter-car). The pavement roughness was described with a filtered white noise model. The rigid foundation was modeled to have a finite-length Euler–Bernoulli beam on a deformable foundation. The top layer was modeled using the assumed modes method. The subgrade was modeled with a Winkler-type foundation, in which the stiffness and damping properties varied along the length. The interaction model was then formulated in an augmented state-space representation. To effectively examine the response of the vehicle, the covariance of the response was then solved for the time-varying Lyapunov equation. Then, the equations were solved for various pavement roughness and foundation cases, to construct the covariance responses. Based on the estimated responses, six features that could distinguish the foundation types were selected and employed on a classifier. Subsequently, noise-added responses were employed on a linear classifier and demonstrated that the measured dynamics of a vehicle due to interaction could distinguish the foundation types and variations with an accuracy of over 90%.

## 2. Model Formulation

### 2.1. Overview

**Figure 1:**
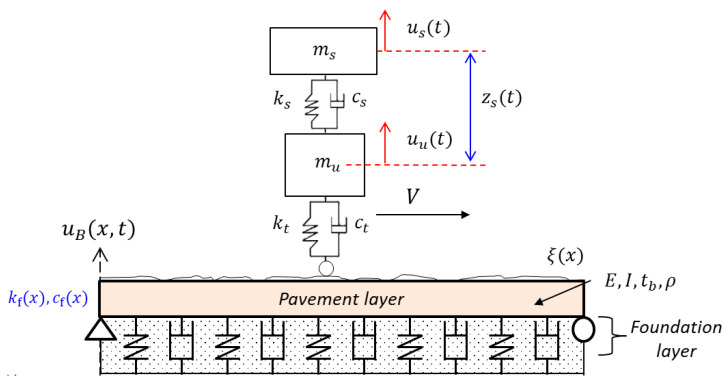
Vehicle–pavement–foundation system.

The vehicle–pavement–foundation interaction model considered herein is shown in Figure 1. The rigid pavement was modeled with an Euler–Bernoulli beam that had constant material properties. Elastic modulus (E), the moment of inertia (I), thickness (*t_b_*), cross-sectional area (*A*), density (ρ), and length (*L*). The vertical displacement of the beam due to interaction was defined as $u_{B}\left(x,t\right).\;$The elastic foundation was taken as a Winkler-type foundation with varying stiffness ($k_{f}\left(x\right)$) and viscous damping ($c_{f}\left(x\right)$), along the length. The roughness of the pavement was modeled as a profile ξ(x) and superimposed on top of the beam.

The vehicle was represented with a quarter-car model, consisting of sprung mass ($m_{s}$) and unsprung mass ($m_{u})$. Their vertical movement was defined as $u_{s}$ and $u_{u}$, while spring stiffness and damping coefficients at suspension and tire were denoted with $k_{s}$, $k_{t}$, $c_{s}$, and $c_{t}$, respectively. The vehicle was assumed to have constant velocity (*V*), as it traveled along the length. In the subsequent sections, a model formulation of the interaction system was introduced. Note that some derivations such as a state-space representation of the roughness were briefly discussed, while more detailed formulations could be found in the related literature [24,35,36].

### 2.2. Basic Equations

Employing the assumed modes method, the vertical deflection of the Euler–Bernoulli beam $u_{B}\left(x,t\right)$ could be defined in a series of sine functions, assuming a simple support boundary condition:

(1) $$\phi \left(n\right)=\sin\left(\frac{n\pi \left(x+L\right)}{L}\right),\;n=1,\;2,\;3,\;\ldots N$$

where *N* is the total number of modes in the shape function. Then, the deflection of the beam could be rewritten as $u_{B}\left(x,t\right)=N_{B}\left(x\right)q_{B}\left(t\right)$. $N_{B}\left(x\right)$ is a mode shape vector containing defined mode shapes (ϕ(n)) and $q_{B}\left(t\right)$ is a time-dependent generalized displacement of the beam. The relationships for the first- and second-time derivative are ${\dot{u}}_{B}\left(x,t\right)=N_{B}\left(x\right){\dot{q}}_{B}\left(t\right)$ and ${\ddot{u}}_{B}\left(\eta ,t\right)=N_{B}\left(x\right){\ddot{q}}_{B}\left(t\right)$.

Then, defining the displacement vector as $x_{c}\left(t\right)={\left[\begin{array}{ccc} q_{B}\left(t\right) & u_{u} & z_{s} \end{array}\right]}^{T}$, the equations of motion for the vehicle–pavement–foundation interaction system could be formulated as follows:

(2) $$M_{c}{\ddot{x}}_{c}\left(t\right)+C_{c}{\dot{x}}_{c}\left(t\right)+K_{c}x_{c}\left(t\right)=-P_{g}+P_{c}\xi \left(t\right)$$

where

(3) $$M_{c}=\left[\begin{array}{ccc} {\left[\int_{0}^{L}\rho AN_{B}^{T}N_{B}dx\right]}_{N\times N} & {\left[0\right]}_{N\times 1} & {\left[0\right]}_{N\times 1}\\ {\left[0\right]}_{1\times N} & m_{s}+m_{u} & m_{s}\\ {\left[0\right]}_{1\times N} & m_{s} & m_{s} \end{array}\right]$$

(4) $$C_{c}=\left[\begin{array}{ccc} c_{f}\left(Vt\right)\int_{0}^{L}N_{B}^{T}N_{B}dx+c_{t}N_{B}^{T}\left(Vt\right)N_{B}\left(Vt\right) & -c_{t}N_{B}^{T}\left(Vt\right) & {\left[0\right]}_{N\times 1}\\ -c_{t}N_{B}\left(Vt\right) & c_{t} & 0\\ {\left[0\right]}_{1\times N} & 0 & c_{s} \end{array}\right]$$

(5) $$K_{c}=\left[\begin{array}{ccc} \int_{0}^{L}\mathrm{EI}N_{B}^{”T}N_{B}^{”}dx+k_{f}\left(Vt\right)\int_{0}^{L}N_{B}^{T}N_{B}dx+k_{t}N_{B}^{T}\left(Vt\right)N_{B}\left(Vt\right) & -k_{t}N_{B}^{T}\left(Vt\right) & {\left[0\right]}_{N\times 1}\\ -k_{t}N_{B}\left(Vt\right) & k_{t} & 0\\ {\left[0\right]}_{1\times N} & 0 & k_{s} \end{array}\right]$$

(6) $$P_{g}={\left[\begin{array}{ccc} {\left[0\right]}_{N\times 1}, & -(m_{s}+m_{u})g, & -m_{s}g \end{array}\right]}^{T}$$

(7) $$P_{c}=\left[\begin{array}{cc} -k_{t}N_{B}^{T}\left(Vt\right) & -c_{t}N_{B}^{T}\left(Vt\right)\\ k_{t} & c_{t}\\ 0 & 0 \end{array}\right]$$

(8) $$\xi \left(t\right)={\left[\begin{array}{cc} \hat{\xi }\left(t\right)\; & \dot{\hat{\xi }}\left(t\right) \end{array}\right]}^{T}\;$$

Note that $N_{B}\left(x\right)$ is short noted as $N_{B}$, except for the case when evaluated at x=Vt. Additionally, $\hat{\xi }\left(t\right)={\xi \left(x\right)|}_{x=Vt}$ $k_{f}\left(Vt\right)={k_{f}\left(x\right)|}_{x=Vt}$, $c_{f}\left(Vt\right)={c_{f}\left(x\right)|}_{x=Vt}$ and *g* is the gravity term.

Then, defining a state vector $x_{T}={\left[\begin{array}{cc} x_{c} & {\dot{x}}_{c} \end{array}\right]}^{T}$ and organizing Equation (2) in a state-space representation, yields:

(9) $${\dot{x}}_{T}\left(t\right)=A_{T}x_{T}\left(t\right)+B_{T}\xi \left(t\right)\;=\left[\begin{array}{cc} {\left[0\right]}_{N_{T}\times N_{T}} & {\left[I\right]}_{N_{T}\times N_{T}}\\ -M_{c}^{-1}K_{c} & -M_{c}^{-1}C_{c} \end{array}\right]x_{T}\left(t\right)+\left[\begin{array}{c} {\left[0\right]}_{N_{T}\times 2}\\ M_{c}^{-1}P_{c} \end{array}\right]\xi \left(t\right)$$

where $N_{T}$ is N+2. The output vector $y_{T}\left(t\right)$ could be defined to contain arbitrary information about the system. In this study, the vehicle responses including displacement and velocity of the unsprung and sprung masses were considered as the output, i.e., $y_{T}\left(t\right)=\left[\begin{array}{cccc} u_{u} & z_{s} & {\dot{u}}_{u} & {\dot{z}}_{s} \end{array}\right]$. Then, the observation and feedthrough matrices yield:

(10) $$y_{T}\left(t\right)=C_{T}x_{T}\left(t\right)+D_{T}\xi \left(t\right)\;=\left[\begin{array}{cccccc} {\left[0\right]}_{1\times N} & 1 & 0 & {\left[0\right]}_{1\times N} & 1 & 0\\ {\left[0\right]}_{1\times N} & 0 & 1 & {\left[0\right]}_{1\times N} & 0 & 1 \end{array}\right]x_{T}\left(t\right)+{\left[0\right]}_{2N_{T}}\xi \left(t\right)$$

### 2.3. Augmented Equations of Motion

This section further arranges the equations derived for the interaction problem in Equation (9) to yield an augmented system in which the primary input is white noise. The white noise input allows a much simpler calculation and the use of stochastic analyses, when compared with manually inputting the roughness profile to the system. Authors in [35,36] constructed a state-space model for the stochastic roughness, when it follows a specified PSD, ($S_{\zeta \zeta }\left(\omega \right)$). In their approach, the transfer function ($H_{\zeta w}\left(\omega \right)$) was approximated using polynomial representation, as below:

(11) $$S_{\zeta \zeta }\left(\omega \right)=H_{\zeta w}^{2}\left(\omega \right)S_{0}$$

where $S_{0}$ is the degree of unevenness and ω is radian per second.

Then, $H_{\zeta w}\left(\omega \right)$ is realized in a state-space model as below to have output vector $y_{f}={\left[\begin{array}{cc} \hat{\xi }\left(t\right) & \dot{\hat{\xi }}\left(t\right) \end{array}\right]}^{T}$, i.e.,

(12) $${\dot{x}}_{f}=A_{f}x_{f}\left(t\right)+B_{f}w\left(t\right)$$

(13) $$y_{f}\left(t\right)=C_{f}x_{f}\left(t\right)$$

where $x_{f}$ is the state vector, $A_{f}$, $B_{f}$, and $C_{f}$ are system, input, and observation matrices, respectively. The output vector is defined to contain the roughness and time derivative term of the roughness, i.e., $y_{f}\left(t\right)={\left[\begin{array}{cc} \tilde{\xi }\left(t\right) & \dot{\tilde{\xi }}\left(t\right) \end{array}\right]}^{T}$. An example of designing a pavement filter using a second-order low-pass filter and polynomial approximation approaches is discussed in detail [35].

Finally, by combining Equations (9), (10), (12), and (13), the augmented state vector was defined as $x_{a}={\left[\begin{array}{cc} x_{T}^{T} & x_{f}^{T} \end{array}\right]}^{T}$. Then,

(14) $${\dot{x}}_{a}=A_{a}x_{a}+B_{a}w\left(t\right)\;=\left[\begin{array}{cc} A_{T} & B_{T1}C_{f1}+B_{T2}C_{f2}\\ 0 & A_{f} \end{array}\right]\left[\begin{array}{c} x_{T}\\ x_{f} \end{array}\right]+\left[\begin{array}{c} 0\\ B_{f} \end{array}\right]w\left(t\right)$$

(15) $$y_{T}=C_{a}x_{a}=\left[\begin{array}{cc} C_{T} & D_{T1}C_{f1}+D_{T2}C_{f2} \end{array}\right]\left[\begin{array}{c} x_{T}\\ x_{f} \end{array}\right]$$

Note that $B_{T1}$ and $B_{T2}$ indicate the first and the second columns of $B_{T}$, respectively. Similarly, $D_{T1}\;$ and $D_{T2}$ correspond to the first and the second columns of $D_{T}$. The equations do not contain the feedthrough terms, implying that the system was strictly proper. In addition, $A_{a}$ and $C_{a}$ were time-dependent matrices, due to the variable foundation coefficients, $k_{f}\left(Vt\right)$ and $c_{f}\left(Vt\right)$.

## 3. Stochastic Vehicle Response

The covariance of the augmented system, $\Gamma_{x_{a}}$, could be determined through a linear differential equation, when the input is a white noise process [37]:

(16) $${\dot{\Gamma }}_{x_{a}}\left(t\right)=A_{a}\left(t\right)\Gamma_{x_{a}}+\Gamma_{x_{a}}\left(t\right)A_{a}^{T}\left(t\right)+2\pi S_{0}B_{a}B_{a}^{T}$$

where $S_{0}$ is the level of the white noise indicating the level of roughness. Solving Equation (16) is beneficial as it does not contain the principal matrix, in which an explicit format of the matrix is generally unknown in time-varying systems.

In the case of uniform foundation, i.e., $k_{f}\left(x\right)=k_{f},{\;c}_{f}\left(x\right)=c_{f}$ with an infinite length beam, the system becomes stationary. Then, assuming that the initial conditions could be described by a random vector, $x_{a}\left(0\right)=x_{a0}$ the initial condition of Equation (16) $\Gamma_{x_{a}}\left(0\right)=\Gamma_{0}$ becomes:

(17) $$\Gamma_{0}=E\left[\left(x_{a0}-\mu_{x_{a0}}\right){\left(y_{a0}-\mu_{x_{a0}}\right)}^{T}\right]$$

where $\mu_{x_{a0}}$ and $\Gamma_{0}$ indicates the mean and the covariance, respectively. If the initial conditions are all deterministic, $\Gamma_{0}=0$. Then, the covariance of the structure responses, $\Gamma_{y}$, is given by:

(18) $$\Gamma_{y}=C_{a}\Gamma_{x_{a}}C_{a}^{T}$$

Further, with a zero-mean white noise being the input to the augmented system in Equation (14), the stationary covariance responses could be obtained by the solution of

(19) $$0=A_{a}\Gamma_{x_{a}}+\Gamma_{x_{a}}A_{a}^{T}+2\pi B_{a}S_{0}B_{a}^{T}$$

which is known as the Lyapunov equation [38]. Note that the equation is linear in unknown covariances and can only examine the moments of the responses under the stationary process.

However, the presented study consisted of a non-uniform foundation, in which the quantities varied over the length of the beam. Thus, the basic assumptions made in Equation (20) was no longer valid. Instead of directly integrating Equation (14), the general covariance response in Equation (16) was solved for $\Gamma_{x_{a}}$, for which the matrix components are described below:

(20) $$\Gamma_{xa}=\left[\begin{array}{ccccccc} \Gamma_{qq} & \Gamma_{qu} & \Gamma_{qz} & \Gamma_{q\dot{q}} & \Gamma_{q\dot{u}} & \Gamma_{q\dot{z}} & \Gamma_{qf}\\ \; & \Gamma_{\mathrm{uu}} & \Gamma_{\mathrm{uz}} & \Gamma_{u\dot{q}} & \Gamma_{u\dot{u}} & \Gamma_{u\dot{z}} & \Gamma_{uf}\\ \; & \; & \Gamma_{zz} & \Gamma_{z\dot{q}} & \Gamma_{z\dot{u}} & \Gamma_{z\dot{z}} & \Gamma_{zf}\\ \; & \; & \; & \Gamma_{\dot{q}\dot{q}} & \Gamma_{\dot{q}\dot{u}} & \Gamma_{\dot{q}\dot{z}} & \Gamma_{\dot{q}f}\\ - & \mathrm{sym} & - & \; & \Gamma_{\dot{u}\dot{u}} & \Gamma_{\dot{u}\dot{z}} & \Gamma_{\dot{u}f}\\ \; & \; & \; & \; & \; & \Gamma_{\dot{z}\dot{z}} & \Gamma_{\dot{zf}}\\ \; & \; & \; & \; & \; & \; & \Gamma_{ff} \end{array}\right]$$

where $q_{B},\;u_{u}$, and $z_{s}\;$ are short noted as **q**, u, and z, respectively. Then, the symbolic covariance matrix, for which the number of variables is $N_{\mathrm{var}}=(N_{a}\times \left(N_{a}+1\right)/2)$ was plugged into Equation (15) to construct $N_{\mathrm{var}}$ distinct differential relationships.

Finally, the desired time-varying covariance responses of the vehicle, $\Gamma_{y_{T}}\left(t\right)={\left[\begin{array}{cccc} \Gamma_{\mathrm{uu}}\left(t\right) & \Gamma_{\mathrm{zz}}\left(t\right) & \Gamma_{\dot{u}\dot{u}}\left(t\right) & \Gamma_{\dot{z}\dot{z}}\left(t\right) \end{array}\right]}^{T}$, was obtained by solving Equation (16) via the time-step integration method embedded in Matlab® (e.g., ode45).

## 4. Illustrative Examples

This section demonstrates the proposed approach by examining the covariance response of a vehicle on a non-uniform foundation. To first validate the solution procedure, steady-state covariance responses were compared by slowing the speed of the vehicle. Then, covariance responses were compared for various pavement scenarios. Finally, covariance response features were selected to classify and examine the foundation properties.

### 4.1. Vehicle and Pavement Model Properties

The properties of the quarter-car model used in the numerical examples are drawn from [39,40] and summarized in Table 1. Note that $k_{t}$ is sought using a calibration index to well approximate the empirical model in [41], on a non-deformable rigid foundation with varying roughness [35].

The transfer function to approximate the PSD of road roughness, as in Equation (11), is considered as follows:

(21) $$S_{\xi \xi }\left(\omega \right)=S_{0}{\left(\frac{\Omega }{\Omega_{0}}\right)}^{-\nu }$$

where Ω is the spatial circular frequency (ω/V); $\Omega_{0}=1\;\mathrm{rad}/m$, ν is the waviness that is taken as 2.45, to match the average roads in the U.S. [42]. Note that $S_{0}$ is varied to match the International Roughness Index (IRIs), which is a measure of road roughness on ride comfort [43]. A lower IRI value indicates a smooth pavement, while a higher IRI implies a rough pavement. In this study, IRIs are varied from 1 to 5, and the corresponding $S_{0}$’s are approximated at those integers, using the golden car approach described in [35].

The typical pavement system was adopted herein, and the uniform properties of the Euler–Bernoulli beam (top layer in Figure 1) are summarized in Table 2. The elasticity of the top-layer used in the study represents the medium soil [44].

**Figure 2:**
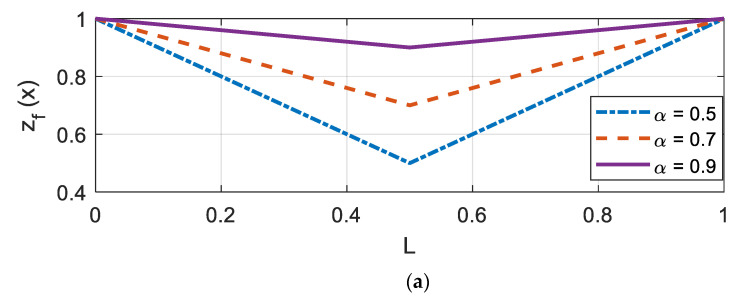

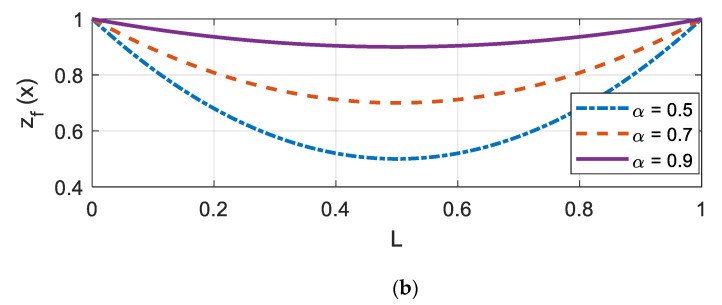
Nonlinear variation in the Winkler foundation (**a**) linear variation; (**b**) quadratic variation.

Finally, to accommodate different Winkler type foundations, spring and damping coefficients were varied linearly or quadratically. Thus, the following equations were adopted for each case:

(22) $$k_{f}\left(x\right)=k_{f0}\times z_{f}\left(x\right)\;c_{f}\left(x\right)=c_{f0}\times z_{f}\left(x\right)$$

(23) $$\mathrm{Linear}\text{:}\;z_{f}\left(x\right)=1-\alpha x,\;0\leq x\leq L/2\;z_{f}\left(x\right)=\left(2\alpha -1\right)-2x\left(\alpha -1\right)/L,\;L/2\leq x\leq L$$

(24) $$\mathrm{Quadratic}:\;z_{f}\left(x\right)=1+4x\left(\alpha -1\right)/L-\left(\alpha -1\right)4x^{2}/L^{2},\;0\leq x\leq L$$

where the reference parameters for stiffness ($k_{f0}$) and damping ($c_{f0}$) are 30 kPa/mm and $2.4\times 10^{7}\;$N·s/m, respectively. The reduction factor α≤1 was selected such that the soil had the most reduced value at the mid-span of the beam (*L*/2). In this study, α was varied from 0.5, 0.7, and 0.9, which implied 50%, 70%, and 90% of the reference parameters. An illustration of $z_{f}\left(x\right)$ for each α is shown in Figure 2. Herein, the variation profiles are described with L and Q, for a linear and quadratic shape, respectively, followed by two digits describing α. For example, a dashed-dot plot in Figure 2a (linear with α = 0.5) was denoted as ‘L50’. Similarly, ‘Q90’ indicates that $z_{f}\left(x\right)$ varies in a quadratic manner (see a solid line in Figure 2b).

### 4.2. Validation of the Solution Approach

In this section, the steady-state responses of the vehicle were compared with that of near-stationary responses. The purpose of the presented study was to validate the time-varying covariance solutions in Equation (16) by slowing the speed of the vehicle, and also to illustrate the difference in the response due to various profiles of the subgrade and roughness, to be used in the following sections.

**Figure 3:**
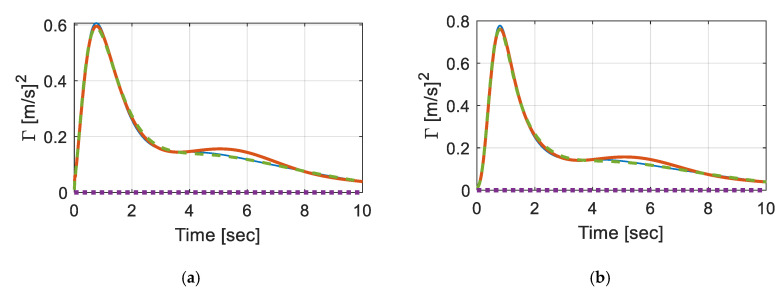

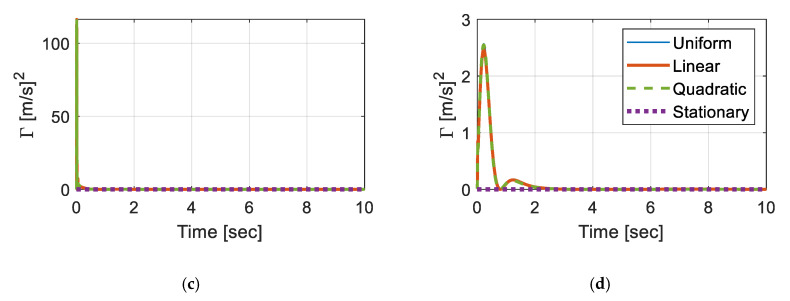
Covariance response of the vehicle over time (**a**) $\Gamma_{u_{u}u_{u}}$; (**b**) $\Gamma_{z_{s}z_{s}}$; (**c**) $\Gamma_{{\dot{u}}_{u}{\dot{u}}_{u}}$; and (**d**) ${\;\Gamma }_{{\dot{z}}_{s}{\dot{z}}_{s}}$.

Figure 3 shows $\Gamma_{y_{T}}\left(t\right)$ in comparison with steady-state responses. Here, the speed of the vehicle was slowed to 0.5 m/s (1.8 km/h). The total number of modes used in the Euler–Bernoulli beam was 10 sine modes and a roughness of IRI = 3 m/km was used. Regarding subgrade, the uniform foundation was represented by $z_{f}\left(x\right)=1$ in Equation (19), while α=0.5 was used for linearly and quadratically varying foundations. Steady-state responses were calculated by fixing the location of the vehicle at the mid-span and solving Equation (16) with ${\dot{\Gamma }}_{x_{a}}\left(t\right)=0$, where the responses were as small as $\Gamma_{u_{u}u_{u}}=1.39\times 10^{-5}\;{\left[m/s\right]}^{2}$; $\Gamma_{z_{s}z_{s}}=1.40\times 10^{-5}\;{\left[m/s\right]}^{2}$; $\Gamma_{{\dot{u}}_{u}{\dot{u}}_{u}}=1.42\times 10^{-5}\;{\left[m/s\right]}^{2}$; $\Gamma_{{\dot{z}}_{s}{\dot{z}}_{s}}=0.189\times 10^{-5}\;{\left[m/s\right]}^{2}$. Note that the impact of foundation property change in the case of a stationary process was negligible, as reported by [24]. However, non-stationary covariance responses showed large humps within the first 2 s, due to the dynamic effect of boundary conditions. Peaks on velocity covariance responses were much higher than the displacement responses, rapidly converging to stationary responses.

**Figure 4:**
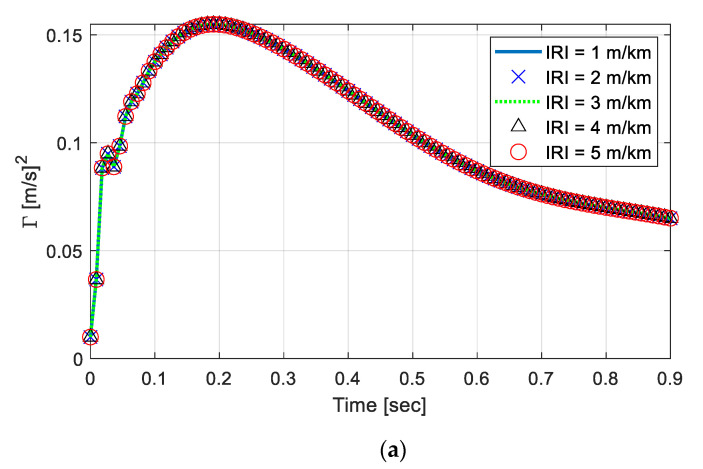

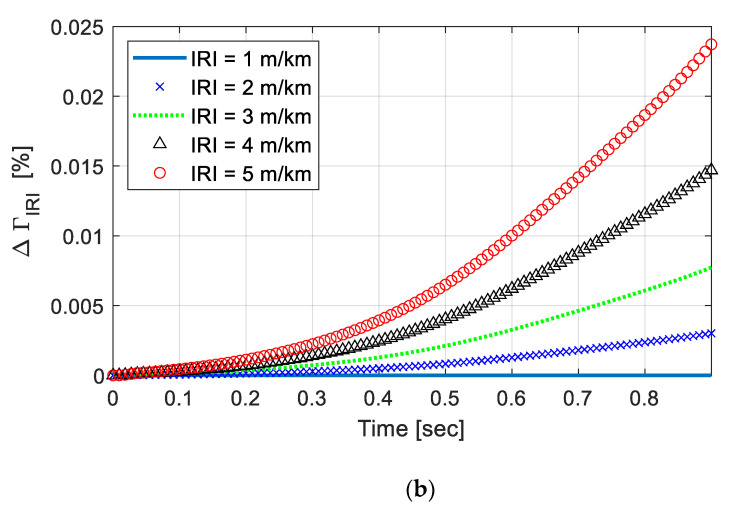
(**a**) Covariance response of vehicle under varying IRIs. (**b**) Difference in the response among IRIs in percentage.

Furthermore, the responses were examined on various road roughness. To compare the results efficiently, Figure 4a plots $\Gamma_{u_{u}u_{u}}$ with linearly varying foundation, as the IRIs varied from 1 to 5 m/km. A time-step integration method, ode45 was used as the vehicle crossed over the length with vehicle speed (V = 20 km/h). As could be seen, the effect of surface roughness on the nonstationary covariance responses were negligible. To better visualize the difference in the responses, the differences were plotted in Figure 4b, in percentage. The difference was estimated for each IRI ($\Gamma_{IRI_{i}}$), with respect to the response at IRI = 1 km/m ($\Gamma_{IRI_{1}}$), as below:

(25) $${\Delta \Gamma }_{IRI}=\left(\frac{\Gamma_{IRI_{i}}-\Gamma_{IRI_{1}}}{\Gamma_{IRI_{1}}}\right)*100\;\left[\%\right]\left(i=1,2,\ldots ,5\right)\;$$

${\Delta \Gamma }_{IRI}$ tended to diverge as the vehicle moved along the beam, indicating that dynamic responses were accumulated. However, within the domain, the maximum difference was less than 0.025% which was negligible, compared to the governing dynamic responses. Although the impact of change in IRI might increase as the beam length gets longer and the speed of the vehicle increases, the result indicated that the responses were governed more by the non-uniform features of the foundation. This fact emphasized the importance of conducting nonstationary response analyses because the stationary response analyses could not capture such differences, as reported by [24].

Based on the study, non-stationary responses under various subgrades converged to steady-state responses with time, while velocity covariance responses showed a faster rate. Additionally, the subgrade variation types affected the vehicle responses while road roughness had a negligible impact. Therefore, in the subsequent sections, road roughness was fixed to IRI = 3 m/km, to examine $\Gamma_{u_{u}u_{u}}\left(t\right)$ on various α’s.

### 4.3. Time-Varying Covariance Responses

**Figure 5:**
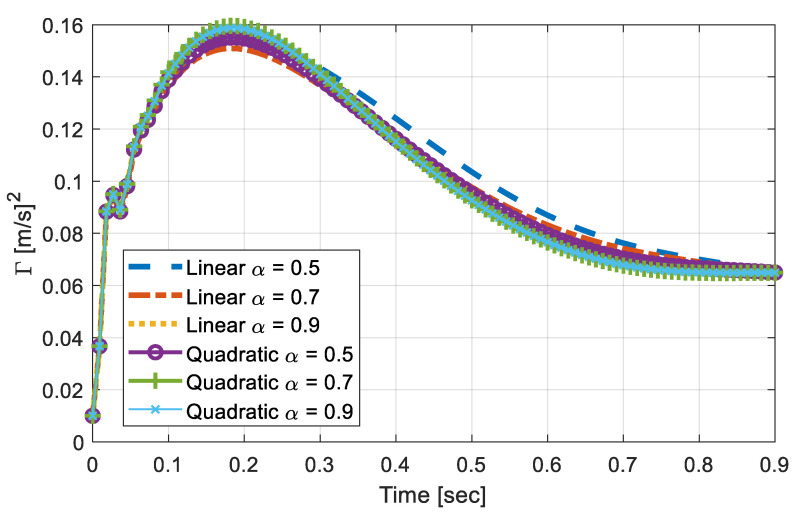
Covariance response on various foundation properties (without noise).

Figure 5 shows the time-varying covariance responses, $\Gamma_{u_{u}u_{u}}\left(t\right)$, as the vehicle runs over the pavement with different types of foundation properties. The simulations were carried out on the basis of combinations of two variables—(1) foundation profile, implying how the subgrade properties change, i.e., either in a linear or in a quadratic manner, and (2) α, which varied from 0.5, 0.7, to 0.9. Then, the variable time-step was used for the integration and then downsampled to present 100 data points within the duration. After the resampling procedure, one could consider that the responses on the vehicle were measured at about 110 Hz. The chosen sampling rate was low enough to be easily realized, yet could capture the key features of the responses. The response shown in Figure 5 are deterministic, as the foundation variation profiles, *α*, and the speed of the vehicle are known. However, deterministic identification based on Figure 5 was unrealistic, because the measured signals tended to be contaminated with noises.

**Figure 6:**
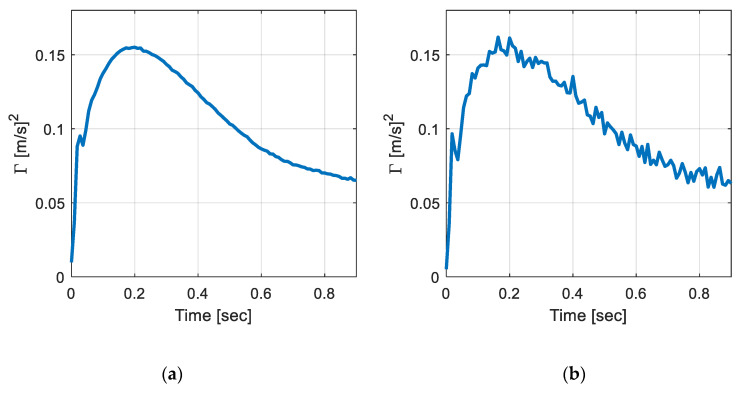
Covariance response with measured noise (**a**) SNR of 45 dB; and (**b**) SNR of 25 dB.

Now, noises were randomly selected to have a signal to noise level (SNR) between 25–50 dB. Note that the noises were added to the raw signals and then resampling was performed to represent the signal noises. Here, only the measured noises were considered because the zero-mean noises in the responses did not affect the covariance responses. For example, Figure 6 illustrates the covariance response with noises added on linearly varying foundation with *α* = 50. SNR of Figure 6a is about 45 dB and Figure 6b is about 50 dB.

From the outcome of this subsection, the following conclusions could be made:

- Variations in profiles and α differed the maximum response and rate of convergence, while a general shape of the responses was preserved.
- A larger maximum value was obtained in quadratically varying profiles, compared to the linear case when the same α was used.
- Within the same profile, a larger α tended to increase the rate of convergence. However, the responses exhibit highly nonlinear relationships between the variables, making the prediction of subgrade’s property change difficult.
- Although the vehicle responses were somewhat deterministic, once the foundation and vehicle parameters were determined, the analytical approach in the prediction was not realistic, due to the noises in the measured signal.

Thus, to resolve the issue, a machine-learning based classification of subgrades based on $\Gamma_{u_{u}u_{u}}\left(t\right)$ is discussed in the subsequent section.

## 5. Machine-Learning Based Classification

Machine-learning techniques are recognized in the civil engineering field as a promising component for monitoring and inspecting [13]. Machine-learning tools can provide pattern recognition strategies, when a deterministic model is difficult to be identified [45]. With their highlighted importance and computational advances, the Matlab software incorporated the Statics and Machine Learning toolbox containing considerable machine-learning techniques [46,47].

Among classifiers provided in the Classification Learner App in Matlab R2019b, one of the traditional classifier, linear discriminant analysis (LDA) is implemented for identifying the changes in the foundation properties from vehicle responses. The LDA method assumes that the data are distributed in Gaussian and that each attribute has the same variance. Then, the Bayes’ theorem is applied to estimate the posterior probability that the observation belongs to a certain class. Then, the costs are evaluated from the maximal difference between the computed sample covariance and the empirical covariance matrix. In LDA, the cost function is linear with respect to the observation [46,48]. With these assumptions, the LDA model attempts to express one dependent variable in terms of a linear combination of other features or measurements [46]. Thus, to enhance the classification accuracy, features in the covariance responses must be selected carefully.

**Figure 7:**
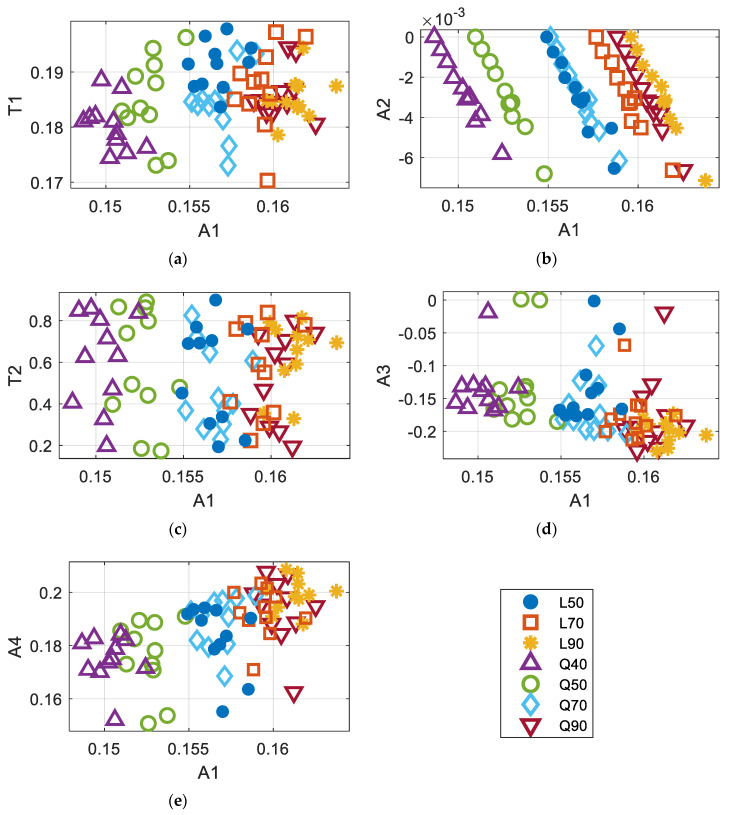
The distribution features over the range of A1 (Maximum Amplitude); (**a**) Relationship between A1-T1 (Maximum Time); (**b**) Relationship between A1–A2 (Maximum Tangent); (**c**) Relationship between A1-T2 (Time at Maximum Tangent); (**d**) Relationship between A1–A3 (Linear regression slope); and (**e**) Relationship between A1-A4 (Linear regression y-intercept).

Based on the previously presented results, the following six features were selected—(1) maximum amplitude, A1; (2) time corresponding to A1, T1; (3) minimum tangent occurring between 0.2 s and 0.8 s, A2; (4) time corresponding to A2, T2; (5) slope of the linear regression between 0.2 s and 0.8 s, A3; and (6) y-intercept of the regression found in (5), A4. Then, to incorporate the measured noise in covariance responses, RMS noises were added in $\Gamma_{u_{u}u_{u}}\left(t\right)$. The extracted features showed some relationships among them. Figure 7 illustrates the distribution of features over the range of A1. As can be seen, some features, such as A2 and A4, show a higher correlation with A1, while other features are more scattered over the range of A1. In addition, L70, L90, and Q90 seem to overlap with each other (as in Figure 7a–e, making it hard to differentiate with classification models that are based on decision trees, etc.

Subsequently, the collected datasets were trained using LDA. The average success rate for using an LDA classifier was over 94%, with at most 10% noise. Table 3 is a confusion matrix when 77 training data were used. The table shows that the foundation was mostly classified, while 18% of the L50 case was misclassified as Q70, and vice versa. Note that LDA showed the highest accuracy when compared with other classification tools; the linear support vector machine showed 82% accuracy, ensemble provided 84% accuracies, while other methods showed over 40% errors.

**Figure 8:**
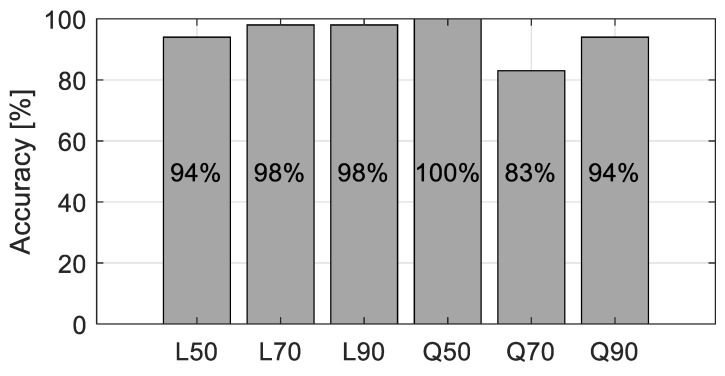
The success rate for identifying foundation property.

Now validation tests were conducted to verify the performance of the developed LDA model. For each case of the foundation, 100 test sets with 1~10% RMS noises added on the responses were generated. The accuracy of the classifier was plotted as a bar chart shown in Figure 8. As could be expected from the confusion matrix, Q70 showed the lowest accuracy, 83%, followed by L50. Overall accuracy was about 94.5% This result supports that by adopting LDA model, the vehicle responses could classify the change in the foundation properties with good accuracies.

## 6. Conclusions

This paper presented a machine-learning-based classification of non-uniform foundation properties using vehicle responses. The dynamics response of the quarter-car model on the stochastic deformable pavement with a finite length was evaluated. A filtered white noise was used to represent the stochastic pavement roughness. The deformable subgrade was modeled by an Euler–Bernoulli beam on a Winkler-type foundation. The non-uniform characteristics were represented with varying stiffness and damping coefficients of the subgrade. Then, the vehicle–pavement–foundation interaction model was combined to yield an augmented state-space representation, which had white noise as the primary input to the system. In this study, the model could accommodate any time of foundation that was describable with a longitudinal axis, although only the impacts of linear and quadratic variations were discussed. A time-varying Lyapunov equation governing the covariance of the responses was solved to effectively obtain the response of the vehicle. From the steady-state Lyapunov solution, the solution approaches were validated. Then, various values of the subgrade’s properties, along with surface roughness were compared. The parametric study showed that the stiffness produced some difference in the response profile, while the roughness produced negligible change. This fact opposed the uniform foundation case, indicating the importance of considering the non-uniform foundation. Then, a set of simulations for measuring noise were performed and used for feature extraction for a classifier. Using an application embedded in Matlab®, linear discriminant analysis was employed to show an average accuracy of 94%. Finally, a total of 600 test sets were generated to demonstrate that the estimated foundation properties were mostly correct. Based on the outcome of this study, the contribution of the presented work and the specific conclusions are summarized as follows:

- The introduced vehicle–pavement–foundation model and nonstationary solution approach allow the investigation of the impact of nonuniform foundation characteristics on vehicle responses.
- Due to the non-stationary stochastic solution approach described, which examined the second-order statistics of the process, efficient estimation was available, where the response was determinate and unaffected by the zero-mean noises.
- The proposed approach could efficiently handle various types of vehicles, roughness, and nonlinearity of foundations.
- Based on the theoretical evaluation, a machine-learning-based classification of non-uniform foundation properties was demonstrated, which included irremovable measured noises.
- In addition to the physical realization of the presented results, future research must ensure to provide high accuracy of identification when the location of the weakened foundation is unknown, and should consider the lateral movement at the left support.
- Overall, based on the outcome of the study, the vehicle responses could be used in conjunction with machine-learning technologies for classifying the properties and types of the subgrade.

In conclusion, the presented work demonstrated the potentials of monitoring the subgrade anomalies from an inspecting vehicle that is only equipped with a set of accelerometers. Unlike the current approach of a subgrade survey that is only limited to suspicious spots, the successful realization of the presented methodology might allow a complete survey and the construction of a database for road management.

## Figures and Tables

**Table 1:** Vehicle properties.

Symbol	Components	Value
$m_{s}$	Sprung mass	1460 kg
$m_{u}$	Unsprung mass	80 kg
$c_{s}$	Suspension Damping	8760 Ns/m
$c_{t}$	Tire Damping	700 Ns/m
$k_{s}$	Suspension Stiffness	29.44 kN/m
$k_{t}$	Tire Stiffness	2500 kN/m
*V*	Velocity	20 km/h (stated otherwise)

**Table 2:** Euler–Bernoulli beam property.

Symbol	Components	Value
h	Thickness	200 mm
b	Width	1.8 m
E	Elastic modulus	8760 Ns/m
ρ	Density	700 Ns/m
*L*	Length	5 m

**Table 3:** Confusion matrix for foundation property test.

Actual Properties	Assessed Properties					
	L50	L70	L90	Q50	Q70	Q90
L50	0.82	0.00	0.00	0.00	0.18	0.00
L70	0.00	1.00	0.00	0.00	0.00	0.00
L90	0.00	0.00	1.00	0.00	0.00	0.00
Q50	0.00	0.00	0.00	1.00	0.00	0.00
Q70	0.18	0.00	0.00	0.00	0.82	0.00
Q90	0.00	0.00	0.00	0.00	0.00	1.00
